# Pretrained models may fail to capture immunological sequences

**DOI:** 10.1038/s42003-026-10695-x

**Published:** 2026-07-20

**Authors:** Jiahao Ma, Hongzong Li, Jian-Dong Huang, Ye-Fan Hu, Yifan Chen

**Affiliations:** 1 Materials Innovation Institute for Life Sciences and Energy, The University of Hong Kong, Futian, China; 2 School of Biomedical Sciences, Li Ka Shing Faculty of Medicine, The University of Hong Kong, Pokfulam, Hong Kong SAR China; 3 The Hong Kong University of Science and Technology, Kowloon, Hong Kong SAR China; 4 Chinese Academy of Sciences (CAS) Key Laboratory of Quantitative Engineering Biology, Shenzhen Institute of Synthetic Biology, Shenzhen Institutes of Advanced Technology, Chinese Academy of Sciences, Shenzhen, China; 5 Clinical Oncology Center, Shenzhen Key Laboratory for Cancer Metastasis and Personalized Therapy, The University of Hong Kong - Shenzhen Hospital, Shenzhen, China; 6 The University of Hong Kong Shenzhen Institute of Research and Innovation, Shenzhen, China; 7 BayVax Biotech Limited, Shatin, Hong Kong SAR China; 8 Shenzhen BayVax Biotech Limited, Shenzhen, China; 9 Departments of Computer Science and Mathematics, Hong Kong Baptist University, Kowloon, Hong Kong SAR China

**Keywords:** Immunology, Computational biology and bioinformatics

## Abstract

Pretrained models, originally developed for vision and textual data, are not a panacea and may fail to fully represent the complexity of sequences in immunological tasks. In studying pretrained immunological sequence modules of a renowned immunogenicity prediction model, pMTnet, we observe that our carefully designed model removing (ablating) the pretrained T cell receptor (TCR) autoencoder in pMTnet can even improve the prediction accuracy. Furthermore, we note the TCR pretraining data, used by pretrained modules within pMTnet, dramatically deviates from a broader and more representative TCR repertoire. Such findings underscore the impacts of the blend of heterogeneous representations and distribution discrepancy in immunological sequences, which necessitate appropriate coordination of different pretrained models and representative databases.

## Abstract (web summary)

Systematic evaluation of pretrained models in T-cell immunogenicity prediction reveals that heterogeneous embedding misalignment and pretraining data biases severely limit their generalization to real-world immunological sequences.

## Introduction

Pretrained models, exemplified by AlphaFold^1,2^ and large protein language models such as ESM^3^, have revolutionized computational biology. By leveraging vast amounts of unlabeled biological sequences (DNA, RNA, and proteins), these models learn rich latent representations that have achieved substantial success in structure prediction, functional annotation, and de novo protein design. The prevailing paradigm suggests that these “foundation models” serve as universal feature extractors, capable of generalizing across diverse downstream tasks with minimal supervision.

However, the efficacy of this paradigm is not guaranteed across all biological domains. The generalizability of pretrained models in immunological studies remains particularly challenging, primarily due to the hyper-variability of immune receptors, such as T cell receptors (TCRs). Unlike conserved protein families, TCRs exhibit extreme sequence diversity in their Complementarity-Determining Regions (CDRs) to recognize a vast array of antigens. In this context, we identify two critical mechanisms where the direct application of pretrained models may falter. Those limitations are often overlooked in the pursuit of end-to-end learning:

1. **Blend of heterogeneous sequences:** The heterogeneity arising when combining embeddings from independently pretrained models (e.g., a TCR encoder and a peptide-MHC encoder). Without explicit and effective alignment, simply concatenating these geometrically distinct latent spaces can degrade performance compared to simpler baselines.
2. **Distribution Discrepancy:** The pronounced mismatch between the limited curated datasets used for pretraining (usually biased towards studied alleles) and the vast, unobserved space of real-world immunological sequences. This discrepancy can lead to algorithmic bias against and poor generalization to unseen variants.

These challenges are exemplified in the T-cell immunogenicity prediction task, which requires modeling the intricate interaction between three distinct entities: the TCR chain, the antigen peptide, and the Major Histocompatibility Complex (MHC) molecule. A common approach, adopted by methods such as pMTnet^4^, involves fusing features extracted by specialized pretrained models. While intuitively appealing, this strategy relies on the assumption that pretrained features are inherently superior to raw sequence representations, regardless of the embedding compatibility (poor compatibility may instead deteriorate the predictive performance).

In this work, we challenge this assumption through a systematic investigation. We first conducted a controlled experiment and observed a striking phenomenon that a model equipped with simple one-hot encoding for TCR sequences (denoted as pMTnet-NoPreCA) outperforms the original pretrained counterpart on both the original pMTnet test dataset and another out-of-distribution (OOD) test dataset we curated. We visualized the embedding landscapes of the two heterogeneous sequence encoders, showing that they occupy distinct regions of the latent space, as expected for encoders with different architectures and objectives, and quantified the downstream impact of this heterogeneity in our ablation. Moreover, we analyzed the distributional properties of widely used TCR pretraining datasets (e.g., TCR-10k) against large-scale observed repertoires (e.g., OTS-1M), revealing significant coverage gaps. Our findings underscore that sophisticated pretraining is not a panacea; rather, rigorous validation of embedding alignment and distribution consistency is a prerequisite for deploying deep learning models in immunological applications.

## Results

Through an ablation study of pretrained immunological sequence models, we explore two concerns in using pretrained models for immunological sequences, ❶ blend of heterogeneous sequences, and ❷ distribution discrepancy.

### Blend of heterogeneous sequences

**Fig. 1:**
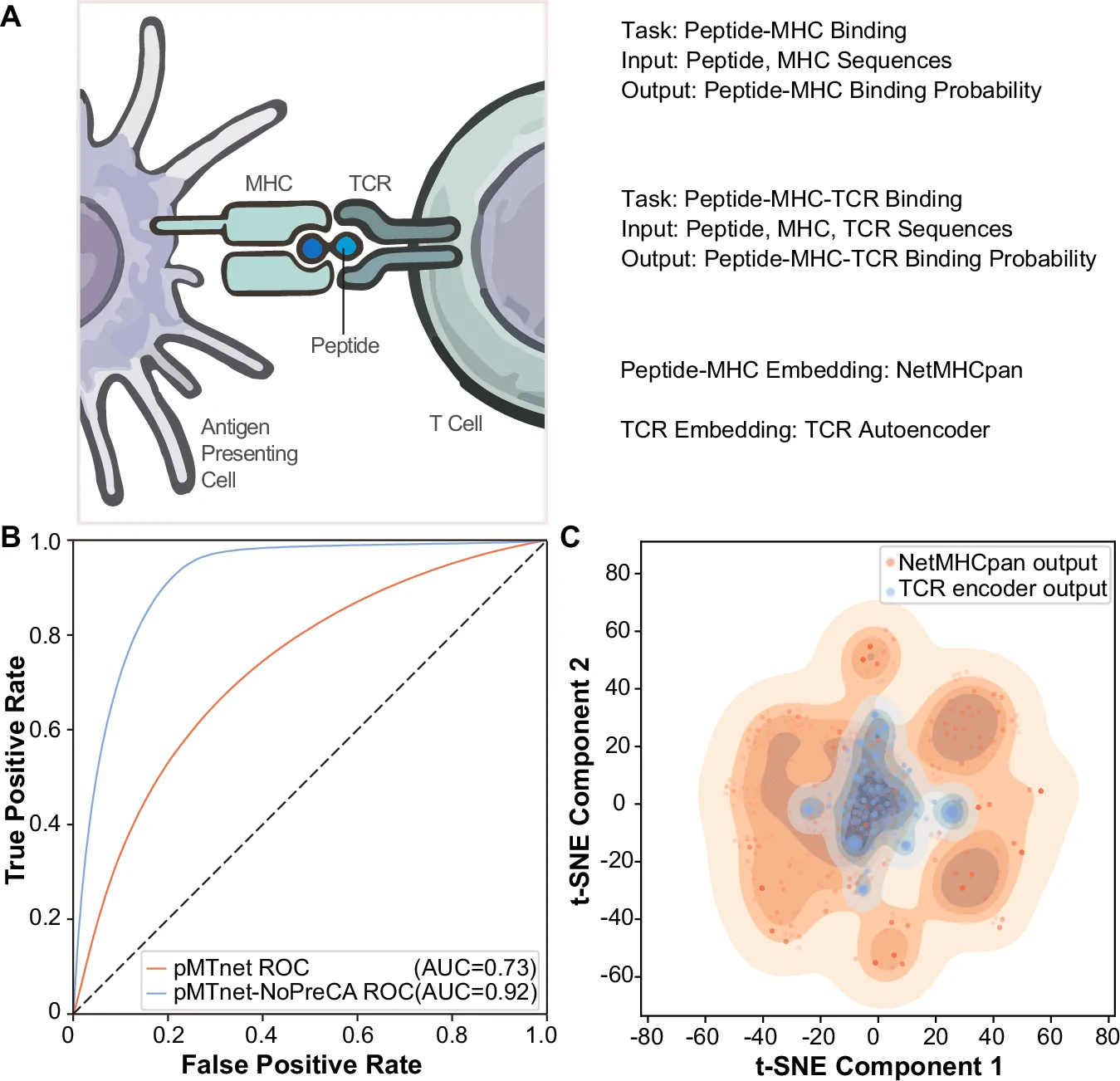
Evaluating T-cell immunogenicity prediction across different sequence blending methods. **A** The immunological process of the T-cell immunogenicity prediction task. **B** The ROC curves on the pMTnet test dataset. pMTnet (the orange curve) concatenates heterogeneous embeddings, while pMTnet-NoPreCA (the blue curve) removes the TCR pretraining, achieving significantly higher ROC AUC. Curves reflect a single training and evaluation run. **C** t-SNE projection of the NetMHCpan (orange) and TCR autoencoder (blue) embeddings computed on the same immunogenicity data. Because the two pretrained encoders have different architectures and training objectives, they occupy distinct regions of the embedding space by construction; this panel illustrates their heterogeneity and is not, by itself, diagnostic of a problem. The quantitative consequence of naively combining such heterogeneous embeddings is established by the ablation in (**B**) and Suppl. Table 1.

Concerns naturally arise when multiple specialized pretrained models produce heterogeneous latent representations in, for example, T-cell immunogenicity prediction task (cf. Fig. 1A). For this task, two pretrained models, TCR autoencoder^5^ and NetMHCpan^6^ (which addresses both peptide and MHC sequences), can be applied to embed the three sequences therein.

However, the corresponding embedding distributions of those two pretrained models substantially differ, even when encoding the same paired immunogenicity data from VDJdb^7^. As shown in Fig. 1C, the TCR autoencoder outputs resemble a normal distribution, whereas the NetMHCpan-derived representations mainly reside on the surface of a ball. While such geometric differences are expected given the encoders’ distinct architectures and objectives, naively concatenating embeddings with such different geometries, without explicit alignment, can adversely affect downstream learning, especially under limited supervision where only a small number of labeled samples are available for task-specific adaptation. We emphasize that this t-SNE visualization is illustrative; the actual performance impact is demonstrated quantitatively by the ablation below.

In this case, the parameters in the predictive model require drastic updates due to the representation misalignment, while the unavoidable lack of application data cannot fulfill the requirements and thus lead to performance drops. Given the pronounced impact of distributional mismatches in embedding spaces (evidenced by improved prediction accuracy after removing the pretrained TCR autoencoder, as shown in Fig. 1B and Suppl. Note 1, “Systematic Comparison of Model Performance”), we conclude that naïvely combining feature representations from biologically distinct tasks or architectural paradigms risks suboptimal model performance.

### Distribution discrepancy

Despite the existence of extensive immunological sequence datasets, only a limited subset of the full sequence space is covered by those datasets. The resulting mismatch between curated datasets and real-world distributions, raises critical concerns about algorithmic bias and model generalizability.

The issue is particularly evident in pretraining for T cell receptors (TCRs), which are protein complexes on the surface of T cells responsible for recognizing fragments of antigen. TCRs are well-known for their high diversity, due to the astronomical rearrangement possibilities of genes (can be as high as 10^23^ theoretically)^8^.

**Fig. 2:**
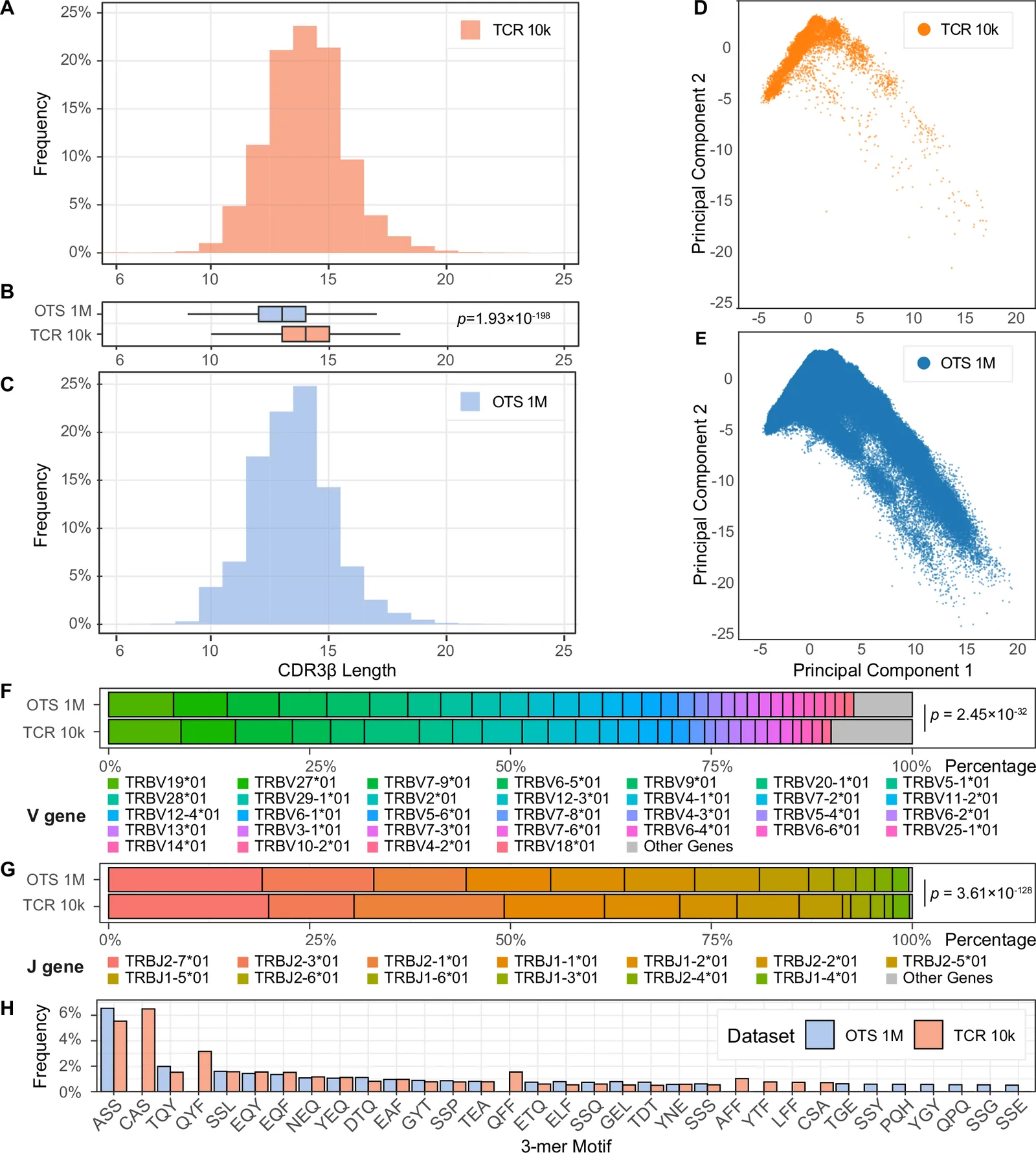
Distribution discrepancy between the TCR 10k dataset (used in pMTnet) and the broader OTS 1M dataset. **A** Frequency distribution of CDR3*β* lengths in the TCR 10k dataset (orange). **B** Boxplot comparison of CDR3*β* lengths between the OTS 1M (blue) and TCR 10k (orange) datasets (two-sided Student’s *t*-test, *p* = 1.93 × 10^−198^). **C** Frequency distribution of CDR3*β* lengths in the OTS 1M dataset (blue). **D** Principal component analysis of amino acid sequence features for the TCR 10k dataset (orange). **E** Principal component analysis of amino acid sequence features for the OTS 1M dataset (blue). **F** V gene usage frequencies for the two datasets (chi-squared test, *p* = 2.45 × 10^−32^). **G** J gene usage frequencies for the two datasets (chi-squared test, *p* = 3.61 × 10^−128^). **H** Frequencies of the most common 3-mer motifs in the two datasets (orange, TCR 10k; blue, OTS 1M).

**Fig. 3:**
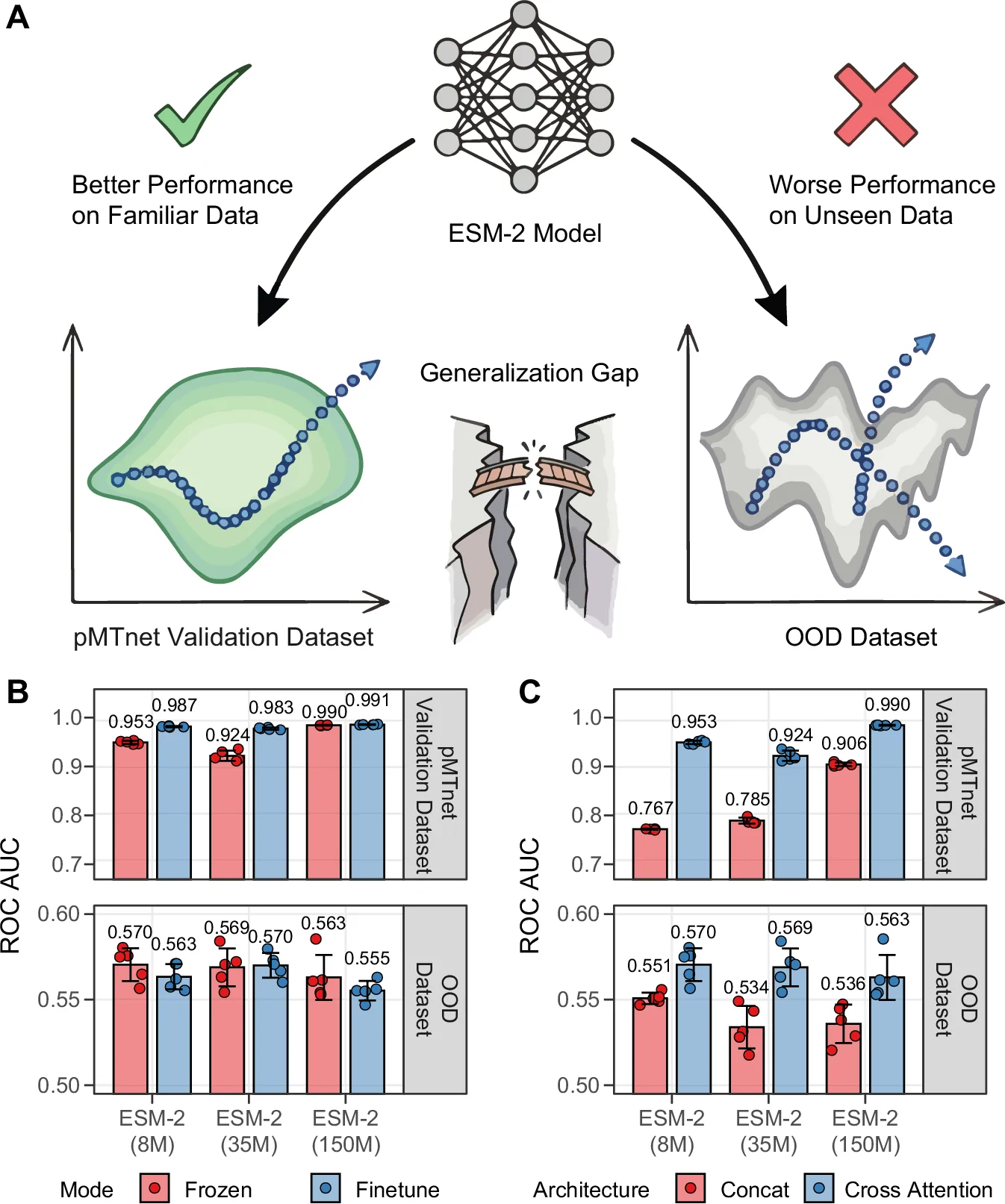
Generalization gap of ESM protein language models on the T-cell immunogenicity prediction task. **A** Schematic of the generalization gap. ESM-2 models achieve high performance on the pMTnet validation dataset but fail to generalize to real-world out-of-distribution (OOD) immunological sequences. **B** Persistent generalization gap across training modes. A sharp disparity between high validation performance and near-random OOD performance remains regardless of whether ESM-2 modules are frozen or finetuned under a cross-attention architecture. **C** Persistent generalization gap across fusion techniques. The performance drop on the OOD dataset remains severe across both concatenation and cross-attention fusion modules (in the frozen setting), indicating that fusion refinements alone do not bridge the generalization gap. In (**B**), red bars denote the Frozen training mode and blue bars the Finetune training mode; in (**C**), red bars denote Concat fusion and blue bars Cross Attention fusion. In (**B**, **C**), bars show the mean, and error bars denote the standard deviation of the ROC AUC over *n* = 5 random seeds (42, 1024, 2024, 7, 0).

In particular, previous studies widely adopted a certain dataset containing  ~ 10,000 TCR sequences (dubbed TCR 10k dataset) to present the entire space of TCR^5^. However, comprehensive profiling reveals systemic biases across multiple dimensions. Specifically, the CDR3 length distribution (Fig. 2A–C) and the V/J gene usage frequencies (Fig. 2F, G) differ significantly between the curated and observed datasets. Furthermore, a principal component analysis (PCA) based on amino acid features (Fig. 2D, E) and 3-mer motif frequency analysis (Fig. 2H) reveal substantial differences between the TCR 10k dataset^5^ and the more representative OTS 1M dataset^9^. This comparison clearly indicates the distribution discrepancy between the pretraining data and the ground truth (cf. Suppl. Fig. 1, Suppl. Table 2, and Suppl. Note 2, “Comparative Analysis of Dataset Distributions”, for a more thorough comparison).

Crucially, the vulnerability to distribution shifts extends beyond specialized TCR models to general-purpose protein language models. In particular, we replace the pretrained modules in pMTnet (the TCR autoencoder and NetMHCpan) with the potent protein language model ESM-2, which is guaranteed not to have seen the OOD test dataset, and re-examine the performance on the preceding T-cell immunogenicity prediction task. The prediction models were trained on the pMTnet training dataset and evaluated on the pMTnet validation dataset (it has the same distribution as the training dataset) as well as the OOD test dataset.

As illustrated in Fig. 3, our evaluation of ESM-2 models reveals a remarkable generalization gap: while achieving near-perfect metrics on the pMTnet validation data, performance collapses on OOD samples unseen in ESM-2 pretraining, regardless of training modes (Fig. 3B) or fusion techniques (Fig. 3C). (Details about the training modes and the fusion techniques we use are provided in the Methods.) This observation confirms that even large-scale foundation models struggle to bridge the distribution discrepancy between pretraining and real-world immunological scenarios.

## Discussion

To handle challenge ❶ heterogeneous sequence data and fundamentally improve performance on diverse tasks, two potential recipes are (1) universal foundation models pretrained on multiple “modalities” (terminology in pretraining studies) of immunological sequences, and (2) potent multimodal fusion techniques that effectively align different modalities. For recipe (1), recent studies have already exhibited empirical successes in handling different immunological sequences with a universal pretrained model^10,11^.

Meanwhile, even without ready multimodal biological foundation models (in recipe (1)) at hand, immunology scientists can alleviate the aforementioned limitation through recipe (2), effective multimodal fusion techniques^12^. As shown in Fig. 1B, we follow the spirit of AlphaFold3 and apply similar fusion techniques to pMTnet-NoPreCA, improving its prediction accuracy. We thus advocate for systematical strategies (such as joint fine-tuning, embedding normalization, contrastive learning, or domain adaptation techniques) rather than direct concatenation of heterogeneous features. In literature, promising architectural approaches include but are not limited to:

1. Co-training frameworks^13^ with weight-sharing constraints that progressively align latent representations.
2. Dual-encoder architectures with adaptive projection layers.
3. Cross-modal attention mechanisms^14^ that learn dynamic alignment weights.

These approaches enable domain-aware calibration of pretrained embeddings through learned transformations that align disparate feature spaces onto compatible manifolds.

For concern ❷, distributional discrepancy is partially attributable to the generation process of common databases. Usually, immunology scientists excessively focus on hotspot sequences, passionately conduct experiments to collect related data, and inadvertently exclude underrepresented cases. Considering the principle of pretraining is to model the distribution of the given database, it is inevitable that representations output by a pretrained model may fail to capture sequence samples deviating from pretraining data. This phenomenon is not unique to TCRs; as shown in Fig. 3A–C, even large protein language models like ESM-2 exhibit a significant generalization gap when applied to out-of-distribution data (ROC AUC  ≈ 0.55), despite high in-distribution accuracy.

Downstream models that build on such biased representations are at risk of further amplifying these disparities, as biases in the output embeddings can cascade into subsequent prediction tasks. More importantly, such underrepresentation leads to bias and ethical dilemmas when models are deployed in clinical settings or large-scale immunological studies. An expedient solution to this issue is that we can limit the predictions to within the subspace covered by the pretraining dataset, especially when constructing baseline models in new clinical applications. However, the community is supposed to make more substantial efforts to uproot the systemic lack of comprehensive datasets. In particular, encouraging active reporting of negative samples in experiments is a cost-effective approach to enhance dataset quality without incurring additional expenses. Moreover, by carefully designing clinical protocols and balancing geographic and demographic variations, data quality can be improved at relatively low costs.

**Fig. 4:**
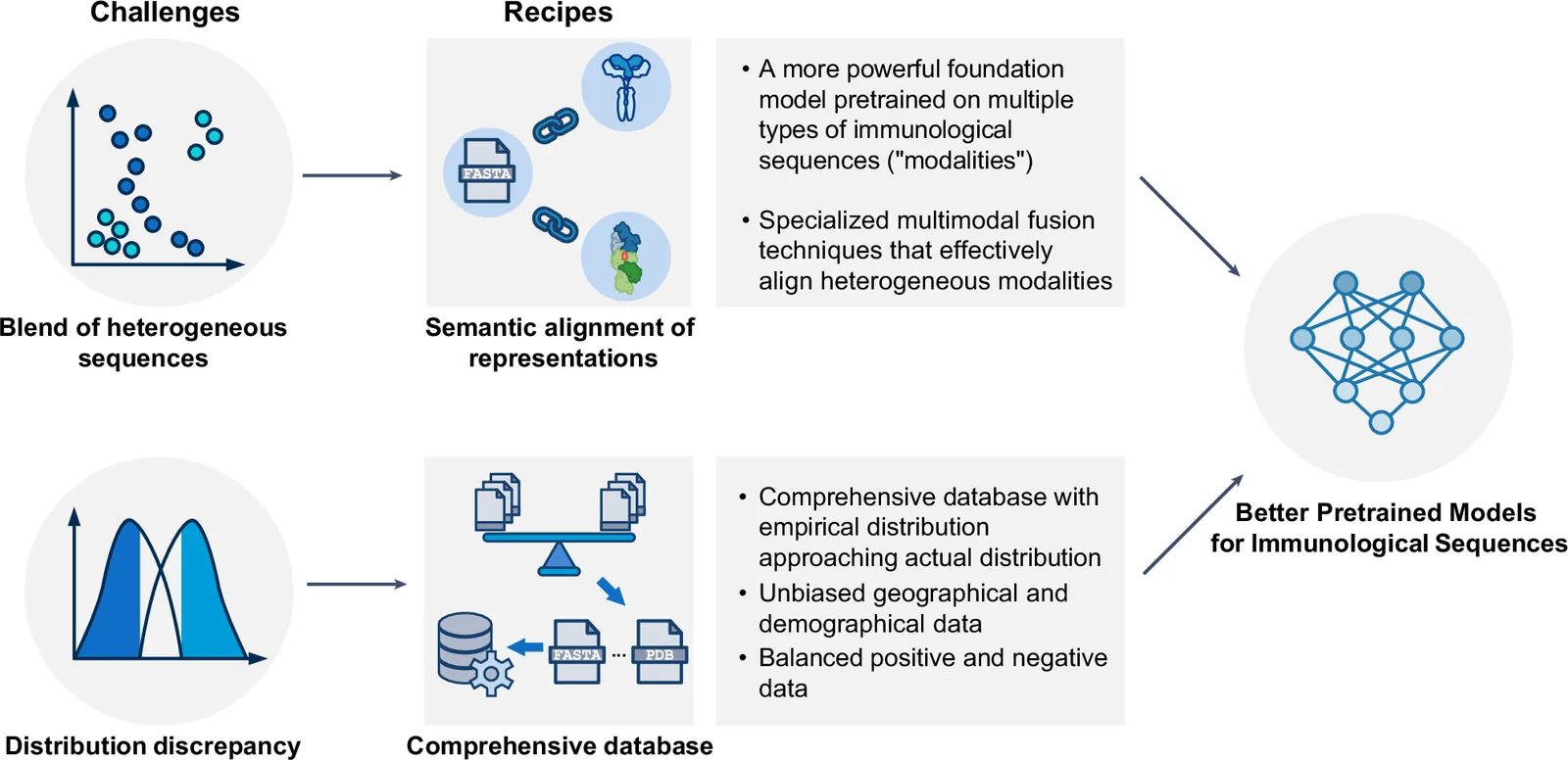
Summary of challenges (heterogeneous sequences, distribution discrepancy) and potential recipes (multimodal fusion techniques, comprehensive databases) for immunological sequence modeling with pretrained models.

The challenges we recognize and the responses we propose are summarized in Fig. 4. In conclusion, the promise of pretrained immunological sequence models hinges on recognizing and remedying potential limitations, from the coarse fusion of heterogeneous sequences to distribution discrepancy in pretraining corpora. With careful inspection of those issues and appropriate application of prediction techniques, we can harness the power of pretrained models without sacrificing the scientific rigor and inclusivity that immunological innovation demands. Furthermore, we appeal to future works on immunological sequences to include rigorous ablation studies, ensuring that pretrained models do provide measurable benefits over task-specific training from scratch. Only through concerted efforts between computational and immunological sciences can we truly unlock the full potential of AI to revolutionize immunological research.

## Methods

This study involves two methods, pMTnet and pMTnet-NoPreCA.

### Models and training

1. Firstly, we reproduce pMTnet^4^, which fuses two pretrained components: a stacked TCR autoencoder (Tessa)^5^ that encodes the CDR3*β* sequence, and a re-implemented NetMHCpan-style pMHC embedding model^6^. We follow the original pMTnet pipeline without modification. Importantly, the peptide–MHC complex is represented not by a binding-affinity score but by a fixed-length numeric embedding: pMTnet extracts the activation vector of an intermediate (pre-output) layer of its re-implemented netMHCpan pMHC model, and reuses this vector directly as the pMHC representation. This pMHC embedding and the Tessa-derived TCR embedding are concatenated and passed to the downstream dense network; no encoder layer is retrained or altered in our reproduction.
2. Secondly, in our proposed model pMTnet-NoPreCA, we remove the original pretrained TCR autoencoder^5^ component, and the TCR sequence encoding is directly utilized as an input to a cross-attention module.
3. Additionally, to evaluate whether the generalization gap extends to general-purpose protein language models, we conduct experiments with ESM-2^15^ at three scales (8M, 35M, 150M parameters). For each scale, we examine two training modes: Frozen (ESM-2 weights fixed, only downstream fusion and classification layers trained) and Finetune (ESM-2 updated end-to-end); and two fusion paradigms: Concat (concatenating mean-pooled embeddings of CDR3, Antigen, and MHC) and Cross Attention (integrating embeddings via multi-head cross-attention). Each configuration was repeated five times with random seeds (42, 1024, 2024, 7, 0).

The hyperparameters listed in Table 1 were chosen based on preliminary experiments and are optimized for the task at hand. The main difference comes from the use of pretrained embeddings in pMTnet.

**Table 1:** Model and training hyperparameters

Model	Hyperparameter	Value
pMTnet	Batch Size	256
	Epochs	150
	Optimizer	Adamax
	Learning Rate	0.002
	Dropout Rate	0.2
	Input Size	90
	FC Layers	300, 200, 100, 2
	Loss Lambda	0.03
	Scheduler	None
	Loss Function	Custom
**pMTnet-NoPreCA**	Batch Size	128
	Epochs	200
	Optimizer	SGD
	Learning Rate	0.01
	Model Dimension	64
	Number of Heads	4
	Input Features	1640
	FC Layers	1024, 1024, 2
	Dropout Rate	0.1
	Loss Function	CrossEntropyLoss
**ESM-2 Experiments**	ESM-2 Scales	8M, 35M, 150M
	Training Modes	Frozen, Finetune
	Fusion Architectures	Concat, Cross Attention
	Downstream Hidden Dim	256
	Number of Heads	4
	Dropout Rate	0.1
	Optimizer	AdamW
	Learning Rate	1 × 10^−^^4^
	Weight Decay	0.01
	Max Epochs	50
	Early Stopping Patience	10
	Batch Size (Frozen)	512 (128 for 150M)
	Batch Size (Finetune)	128 (64 for 150M)
	Random Seeds	42, 1024, 2024, 7, 0

### Dimensionality reduction

For the dimensionality reduction within the experiments in Fig. 2, both datasets (OTS-1M and TCR-10k) were processed consistently using the identical hyperparameter settings (see the details in Table 2). Specifically, Principal Component Analysis (PCA) was performed on both amino acid sequence feature datasets using the same preprocessing steps, including standardization, and the number of principal components was uniformly set to two. Similarly, t-distributed Stochastic Neighbor Embedding (t-SNE) was applied to the output vectors from both models, and a fixed random seed was used to ensure comparability and consistency across results.

**Table 2:** Parameters for PCA and t-SNE analysis

Method	Parameter	Value
PCA	Data type	Amino acid sequence feature vectors
	Preprocessing	Standardization
	Number of components	2
	Explained variance	>80%
	Algorithm	SVD
t-SNE	Input data	Model output vectors
	Target dimensions	2
	Perplexity	30
	Learning rate	200
	Iterations	1000
	Distance metric	Euclidean
	Optimization	Gradient descent
	Random seed	42

### Data sources

The major dataset used in this study was collected from Lu et al.^4^ whose GitHub repository is available at https://github.com/tianshilu/pMTnet. This dataset simultaneously includes TCR-beta CDR3 sequences, peptide sequences, and MHC alleles, which were used to train and validate the performance of the pMTnet model. The dataset is structured to reflect real-world scenarios in T-cell immunogenicity prediction task, with curated examples derived from both experimental and synthetic sources. It involves three sub-datasets: the pMTnet training dataset, pMTnet validation dataset, and pMTnet test dataset.

Similarly, we curated an out-of-distribution dataset (designated as OOD test dataset) by extracting non-overlapping records from VDJdb^7^ via its webserver at https://vdjdb.cdr3.net/on 8 August, 2024. This dataset was specifically curated by removing all sequence duplicates present in the data from Lu et al.^4^. We note that, although the OOD test set is held out and non-overlapping with the training data, it is itself drawn from the curated VDJdb database and therefore represents a curation- and time-separated hold-out rather than a fully independent sample of the real-world TCR repertoire.

For the ESM-2 generalization experiments (Fig. 3), the models were trained on the same training set from Lu et al.^4^. The pMTnet validation dataset was employed to monitor in-distribution performance, and the OOD test dataset served as the held-out test set to evaluate out-of-distribution generalization.

Also, we included paired TCR sequences from Observed TCR Space (OTS), which is available at https://opig.stats.ox.ac.uk/webapps/ots/ots_paired/. This dataset includes  ~ 1,400, 000 TCR CDR3 sequences with empirical distribution more similar to the actual population distribution.

The input data format required by pMTnet includes three essential columns: CDR3, Antigen, and MHC. These represent the TCR-beta CDR3 sequence, peptide sequence, and the corresponding MHC allele, respectively. The processing of these sequences is crucial for the model to learn the binding specificity relationships between TCRs and pMHC complexes.

### Statistics and reproducibility

No statistical method was used to predetermine sample size, and no data were excluded from the analyses. The pMTnet versus pMTnet-NoPreCA comparison (Suppl. Table 1) reflects a single training and evaluation run on the fixed pMTnet datasets, with pMTnet used as the originally published, pretrained model. The ESM-2 experiments (Fig. 3) were each repeated with five random seeds (42, 1024, 2024, 7, 0); bars and error bars denote the mean and standard deviation across these *n* = 5 independent runs. CDR3*β* length distributions (Fig. 2A–C, Suppl. Fig. 1, Suppl. Table 2) were compared with a two-sided Student’s *t*-test (*p* = 1.93 × 10^−^^198^). V and J gene-usage frequencies (Fig. 2F, G) were compared with a chi-squared test (V gene: *p* = 2.45 × 10^−^^32^; J gene: *p* = 3.61 × 10^−128^). TCR repertoire diversity was quantified by the average intra-dataset Levenshtein distance of CDR3*β* sequences over 10 sampling iterations (*n* = 1000 sequences each) and assessed with a binomial test (*p* = 0.00195). Sample sizes and error definitions are also reported in the corresponding figure legends.

## Supplementary information

Supplementary Information

Description of Additional Supplementary Materials

Supplementary Data 1

## Data Availability

The pMTnet training, validation and test datasets were obtained from the original study and its repository at https://github.com/tianshilu/pMTnet^4^. The out-of-distribution test set was curated from VDJdb (https://vdjdb.cdr3.net/), accessed on 8 August 2024^7^. Paired TCR sequences were obtained from the Observed TCR Space (OTS) database (https://opig.stats.ox.ac.uk/webapps/ots/ots_paired/)^9^. The source data underlying all main-text figures are provided as Supplementary Data 1. All other data are available from the corresponding author upon reasonable request.

## Code availability

The custom code used to reproduce the analyses in this study is available at https://github.com/Ascaris-Equi/PretrainHurt and archived on Zenodo at https://doi.org/10.5281/zenodo.20589389^16^. Analyses were performed in Python; key dependencies and their versions are listed in the repository.
