# Supplementary material for: Pretrained models may fail to capture immunological sequences
Source: Commun Biol. 2026 Jul 20;9:1499. doi: 10.1038/s42003-026-10695-x (PMC13752265; doi:10.1038/s42003-026-10695-x)
Supplement: Supplementary file 1 [file 42003_2026_10695_MOESM1_ESM.pdf]

Supplementary Note 1: Systematic Comparison of Model Performance

We provide a thorough comparison of the evaluation metrics for the pMTnet and pMTnet-NoPreCA models, as summarized in **Supplementary Table** 1, which highlights the performance improvement. In addition to the pMTnet test dataset in Lu et al. [4], we generate an additional out-of-distribution dataset (dubbed OOD test dataset) with data from VDJdb [7] using data posted after the publication of Lu et al. [4].

The pMTnet-NoPreCA variant demonstrates superior performance in all assessed categories across both test datasets. On the pMTnet test dataset, pMTnet-NoPreCA demonstrated superior performance, achieving 0.9168 ROC AUC (vs 0.7327 for pMTnet) and 0.8912 average precision (vs 0.7065). This trend persisted across all metrics, with particularly notable improvements in recall (0.9439 vs 0.7504) and the Matthews correlation coefficient (MCC) (0.7210 vs 0.3473). More critically, pMTnet-NoPreCA maintained robust out-of-distribution (OOD) performance, substantially outperforming pMTnet on the OOD test dataset (ROC AUC: 0.7126 vs 0.4722; F1 score: 0.6795 vs 0.3406). The MCC metric most dramatically illustrated this OOD advantage, where pMTnet-NoPreCA achieved positive discrimination (0.3065) compared to near-random performance from pMTnet (-0.0218).

Two caveats apply to this comparison. First, pMTnet and pMTnet-NoPreCA differ not only in the TCR encoding (pretrained autoencoder versus one-hot) but also in the fusion architecture, optimizer, and loss function (**Table** 1); the comparison therefore isolates the effect of the pretrained TCR component only approximately, rather than as a strictly single-variable ablation, although the consistent improvement across all metrics and both test datasets indicates that the pretrained TCR autoencoder is not necessary for, and is here detrimental to, predictive performance. Second, unlike the ESM-2 experiments, which were repeated over five random seeds, the pMTnet versus pMTnet-NoPreCA comparison reflects a single training and evaluation run on the fixed pMTnet datasets, and the reported differences should be interpreted accordingly.

Supplementary Table 1: Evaluation metrics for pMTnet and pMTnet-NoPreCA on the pMTnet test dataset and the OOD test dataset.

	**pMTnet Test Dataset**		**OOD Test Dataset**	
**Metric**	**pMTnet**	**pMTnet-NoPreCA**	**pMTnet**	**pMTnet-NoPreCA**
ROC AUC	0.7327	0.9168	0.4722	0.7126
Average Precision	0.7065	0.8912	0.4845	0.7031
Accuracy	0.6715	0.8547	0.4903	0.6508
Precision	0.6481	0.8011	0.4822	0.6278
Recall	0.7504	0.9439	0.2633	0.7405
F1 Score	0.6955	0.8666	0.3406	0.6795
MCC	0.3473	0.7210	-0.0218	0.3065
PR AUC	0.7065	0.8912	0.4832	0.7031

Supplementary Note 2: Comparative Analysis of Dataset Distributions

We first illustrate the differences between the OTS 1M and TCR 10k datasets via the lens of the sequence length distribution of CDR3 regions (see **Supplementary Figure** 1 and **Supplementary Table** 2). While both datasets spanned a broad length range (OTS: 5-83 aa; TCR: 1-25 aa), their central tendencies diverged significantly. The TCR 10k repertoire exhibited longer median CDR3 lengths (14 aa vs. 13 aa in OTS) with the same interquartile range (13-15 aa vs. 12-14 aa in OTS; both have an interquartile range of 2).

Extreme length outliers were particularly notable in the OTS dataset, which contained CDR3 sequences up to 83 amino acids — more than triple the maximum length observed in the TCR 10k (25 aa). This expansion at the upper length range contributed to the OTS library's mean (13.49) being notably higher than its median (13), reflecting right-skewness, whereas the TCR 10k dataset shows a more symmetric distribution (mean 14.02 vs. median 14). Statistical comparison revealed this mean difference is highly significant (Student's t-test: $p = 1.93 \times 10^{-198}$). Overall, the TCR 10k dataset shows a more symmetric distribution between 9-20 amino acids (median 14 vs. mean 14.02), while the OTS 1M dataset exhibits a slightly right-skewed distribution (median 13 vs. mean 13.49).

Supplementary Table 2: Comparative length distribution analysis of CDR3 regions between OTS and TCR datasets.

	Min.	1st Qu.	Median	Mean	3rd Qu.	Max.
OTS 1M	5	12	13	13.49	14	83
TCR 10k	1	13	14	14.02	15	25

Lastly, we compare **TCR repertoire diversity** between two datasets (TCR-10k and OTS-1M) using averaged Levenshtein distance of intra-dataset CDR3β sequences. With 10 iterations of sampling (n=1000), it consistently shows higher Levenshtein distance among sequences in OTS-1M (mean: 9.003) versus TCR-10k (mean: 8.385), with all the 10 mean differences positive ($p = 0.00195$ by the binomial test). The results indicate significantly greater sequence diversity in the OTS-1M dataset.

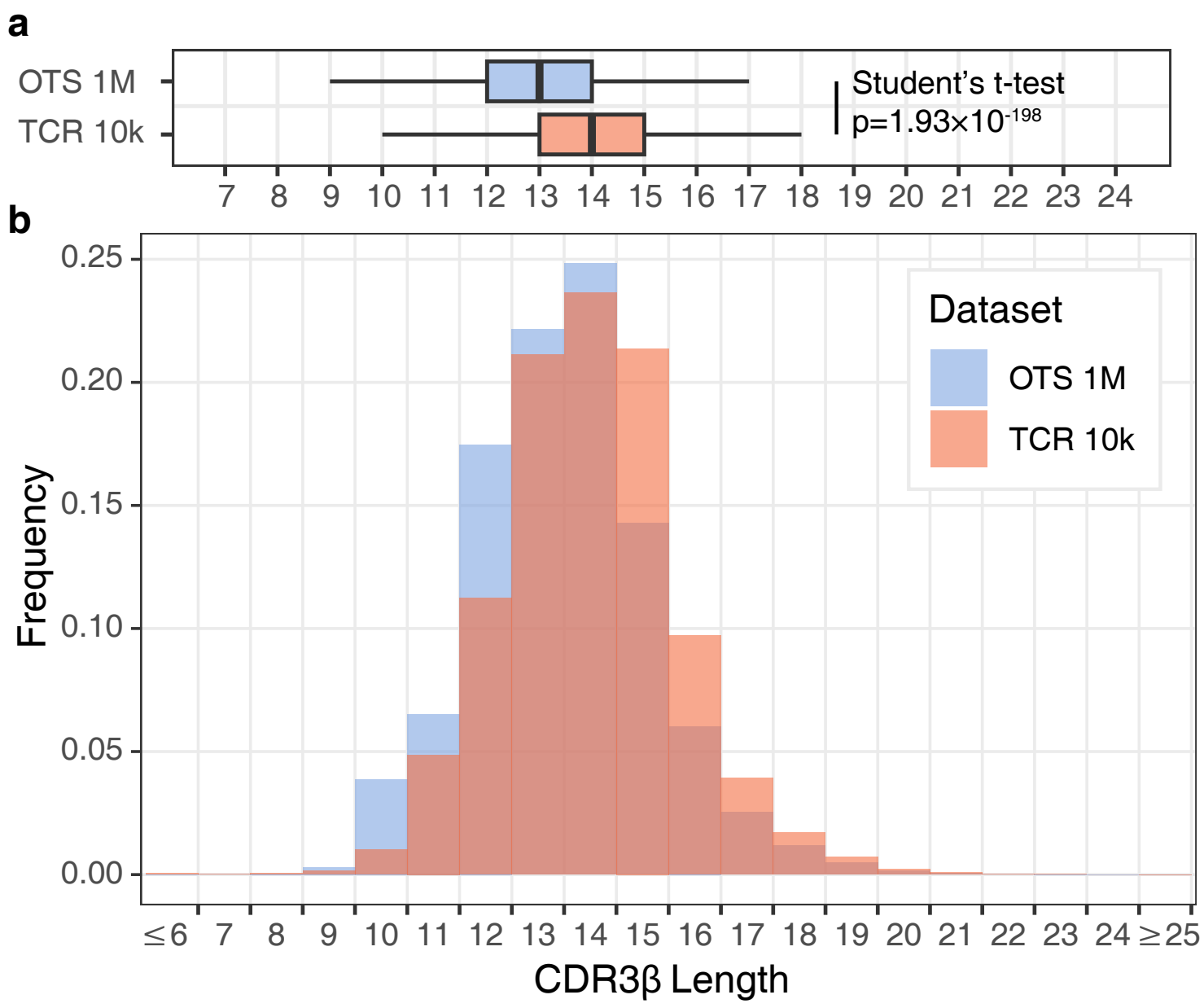

Supplementary Figure 1: Comparative length distribution analysis of CDR3 regions between OTS and TCR datasets. (A) The boxplot of the two datasets analyzed showing significant divergence between datasets (Student's t-test: $p = 1.93 \times 10^{-198}$). (B) Frequency distribution of CDR3β lengths. The OTS 1M library (blue) displays a broader length distribution compared to the TCR 10k repertoire (orange).
