# Supplementary material for: Pretrained models may fail to capture immunological sequences
Source: Commun Biol. 2026 Jul 20;9:1499. doi: 10.1038/s42003-026-10695-x (PMC13752265; doi:10.1038/s42003-026-10695-x)
Supplement: Supplementary file 2 [file 42003_2026_10695_MOESM2_ESM.pdf]

Description of Additional Supplementary Files

File name: Supplementary Data 1

Description: Numerical source data underlying the graphs and charts in the main article figures.
